# Perventricular device closure of a doubly committed juxtaarterial ventricular septal defect through a left parasternal approach: midterm follow-up results

**DOI:** 10.1186/s13019-015-0376-9

**Published:** 2015-11-26

**Authors:** Li Hongxin, Guo Wenbin, Fei Liang, Hai-Zhou Zhang, Mei Zhu, Wen-Long Zhang

**Affiliations:** 1 Department of Cardiovascular Surgery, Shandong Provincial Hospital Affiliated to Shandong University, No. 324 Jingwu Road, Jinan, 250021 China; 2 Echocardiography Lab, Shandong Provincial Hospital Affiliated to Shandong University, No. 324 Jingwu Road, Jinan, 250021 China

**Keywords:** Congenital defects, Minimally invasive, New techniques, Echocardiography, Device

## Abstract

**Background:**

It is infeasible to occlude a doubly committed juxtaarterial ventricular septal defect (DCVSD) percutaneously. The previous perventricular device closure technique was performed through an inferior median sternotomy approach. The purpose of this study is to evaluate the feasibility, safety and efficacy of perventricular device closure of DCVSDs through a left parasternal approach.

**Methods:**

Sixty-two patients, with the DCVSD of less than 6 mm in diameter, were enrolled in this study. The pericardial space was approached through a left parasternal mini-incision without entering into the pleural space. Two parallel pursestring sutures were placed on the right ventricular outflow tract for puncture. Under transesophageal echocardiographic guidance, a new delivery sheath loaded with the device was inserted into the right ventricle and advanced through the defect into the left ventricle. The device, connected with a device stay suture, was deployed subsequently.

**Results:**

Successful device closure of the defects was achieved in 58/62 patients (94 %). The DCVSD failed to close in 4 (6 %) patients due to device-related aortic regurgitation and device migration. The mean DCVSD diameter was 3.4 ± 1.0 mm (range, 2.0 to 6.0 mm). The implanted device size was 5.2 ± 1.3 mm (range, 4 to 8 mm). Forty-four out of 58 patients (76 %) was implanted with an eccentric occluder. The mean intracardiac manipulation time was 14 ± 13 min (range, 2 to 60 min). The procedure time was 66 ± 15 min (range, 42 to 98 min). During the follow-up period of 180 to 1860 (median 880) days, new mild pulmonary regurgitation occurred in 2 patients. No other device-related complications were found. The complete closure rate was 95 % at discharge, 98 % at 1-, 6- and 12-month, 96 % at 2-year, and 100 % at 3-year follow-up.

**Conclusions:**

Perventricular device closure of a DCVSD through a left parasternal approach is feasible, safe, and efficacious in selected patients. This minimally invasive technique permits easy defect crossing and accurate device positioning.

## Background

Percutaneous closure of ventricular septal defects (VSDs) using Amplatzer septal occluders has been shown to be safe and efficacious. It is generally accepted that perimembranous and muscular VSDs can be occluded with excellent results [1–3]. However, device closure of a doubly committed juxtaarterial ventricular septal defect (DCVSD) is difficult to succeed in percutaneous approach because of the specific anatomy of the defect, which is located at the infundibular septum. No reports have been published on percutaneous device closure of DCVSDs until now.

In recent years, perventricular device closure of perimembranous VSDs has been developed and applied clinically with good results [4–7]. However, fewer reports have been published on perventricular device closure of a DCVSD, especially the midterm follow-up results. Moreover, the previous technique was performed through an inferior median sternotomy approach [8, 9]. The lower half of the sternum has to be split open for exposure the puncture site at the right ventricular outflow tract. Operative trauma is still remarkable. Postoperative pain and the pectus carinatum deformity are common after this procedure.

The purpose of this study is to evaluate the feasibility, safety and efficacy of using a new minimally invasive technique to occlude the DCVSDs perventricularly through a left parasternal approach.

## Methods

### Patients’ clinical details

Between May 2009 and November 2014, 62 patients underwent perventricular device closure of DCVSDs through a left parasternal approach in our hospital. Baseline noninvasive data were obtained by physical examinations, electrocardiography, transthoracic echocardiography (TTE), and chest radiography. The DCVSD was defined as a defect that located at the 12:30 to 2 o’clock position in the parasternal short-axis view on TTE [10]. Fifteen patients had symptoms of recurrent respiratory infection, palpitations, or exercise intolerance. Sixteen patients had a trivial or mild aortic regurgitation. The DCVSD coexisted with a mirror image dextrocardia in 1 patient.

According to their age, the patients were divided into two groups: 35 patients younger than 5 years were included as a younger age group and the remaining 27 were included as an older age group.

Indications for DCVSD closure were the same as those used for surgical closure, which included hemodynamically significant left to right shunts, left ventricular chamber enlargement, and (or) mild to moderate pulmonary hypertension. The inclusion criteria for device closure of DCVSDs included: 1) age of 6 months or older; 2) a maximum diameter of the DCVSD of less than 6 mm and 3) left to right shunt. Exclusion criteria included patients with the defect size of more than 6 mm, aortic valve prolapse, moderate or severe aortic regurgitation, intracristal muscular VSD in the outlet septum, contraindications to antiplatelet therapy, and those coexisting with other cardiac anomalies. The enrolled patients or their guardians hoped to close the defect, eliminate the heart murmur, reduce the risk of aortic prolapse, and have a cosmetic procedure. Once a patient met the enrollment criteria, he/she or the guardian was fully informed of the available treatment options. Informed consent was obtained from each patient or from his or her parents. The study was approved by the ethics committee of our institution and performed in compliance with the institutional guidelines and those of the American Physiological Society. The baseline characteristic data of both groups are shown in Table 1.

**Table 1:** Clinical data and outcome for the 58 successful patients

Variable	Total	Younger age group	Older age group	*p* value
Patients’ number	58	32	26	—
Median age (yrs)	4.0 (range, 0.5–53.0)	2.0	12.0	—
Sex (F/M)	15/43	7/25	8/18	—
Median weight (kg)	19 (range, 6–80)	13	39	—
DCVSD size (mm)	3.4 ± 1.0(range, 2.0–6.0)	3.2 ± 0.9	3.6 ± 1.2	—
LVEDD before operation (cm)	3.8 ± 0.8(range,1.9–5.6)	3.2 ± 0.4	4.5 ± 0.5	—
LVEDD at discharge (cm)	3.5 ± 0.7(range,1.8–5.2)^a^	3.0 ± 0.4	4.1 ± 0.6	0.073^a^
Device size (mm)	5.2 ± 1.3 (range, 4–8)	5.0 ± 1.0	5.3 ± 1.5	—
ICMT (min)	14 ± 13 (range, 2–60)	12 ± 8	17 ± 17	0.157
Procedure time (min)	66 ± 15 (range, 42–98)	59 ± 11	74 ± 16	0.0004
Eccentric occluder (n)	44	26	18	0.450
Concentric occluder (n)	14	6	8	—
Drainage tube placement (n)	19	8	11	0.265

*DCVSD* doubly committed ventricular septal defect; *LVEDD* left ventricular end-diastolic diameter; *ICMT* intracardiac manipulation time

^a^compared with the LVEDD before operation

### Device and delivery system

The closure device used in this study was a modified double-disk occluder (Starway Medical Technology, Inc. Beijing, China), based on the Amplatzer septal occluder. There were two types of devices used in this study: the concentric occluder with the left disk 2 mm larger than the connecting waist and the eccentric occluder with the left disk exceeding the connecting waist by 0 mm in its superior part and by 4.0 mm in its inferior part. The right disk is 2 mm larger than the waist in both devices. The waist of the modified occluder, which is 3 mm in length, is longer than that of the Amplatzer occluder.

**Fig. 1:**
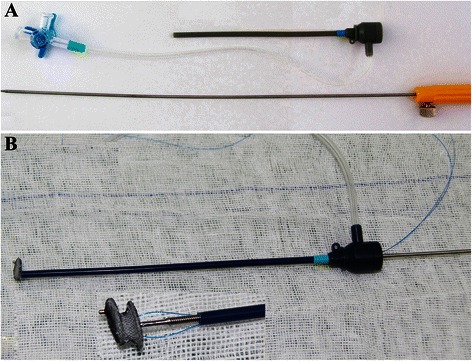
**a** Direct delivery system. **b** The eccentric occluder with a device stay suture (magnification of inset 2.5×) was adjusted to keep the platinum marker on the left disk towards the sheath’s side arm

The new delivery system, which is called direct delivery system (DDS), consists only of a short delivery sheath with a side arm for removal of air and a delivery cable. The delivery sheath is about 10 to 15 cm long, ranging from 5 to 10 F in size (Fig. 1a).

### Transesophageal echocardiography (TEE) and device selection

A PHILIPS IE33 echocardiography instrument (Philips Healthcare, Best, The Netherlands) with a 2.0 to 7.0 MHz frequency conversion probe was used.

After general anesthesia and intubation, patients were placed in a supine position. The defect size and the margins adjacent to the aortic valve (the subaortic rim) and pulmonary valve were measured in the left ventricular long-axis view, 5-chamber view and short-axis view on TEE. The integrity of the aortic and pulmonary valve was assessed simultaneously.

The device selection is determined according to the size and the subaortic rim of the defect. If the subaortic rim is at least 1 mm, a concentric occluder was tried first with the size 1 mm larger than the maximum size of the defect. If the subaortic rim is less than 1 mm, an eccentric occluder is selected with the size 2 to 3 mm larger than the maximum diameter of the defect.

### Procedure

The DCVSDs were occluded perventricularly using the DDS. The selected device was screwed onto the delivery cable and pulled inside the delivery sheath under water with the tip extruded out of the sheath. A device stay suture of 4–0 or 5–0 polypropylene (Ethicon, Somerville, NJ) was passed through the wire mesh of the device under the microscrew and pulled out of the sheath. The eccentric occluder was adjusted to keep the platinum marker on the left disk towards the side arm before it was retracted into the sheath (Fig. 1b).

**Fig. 2:**
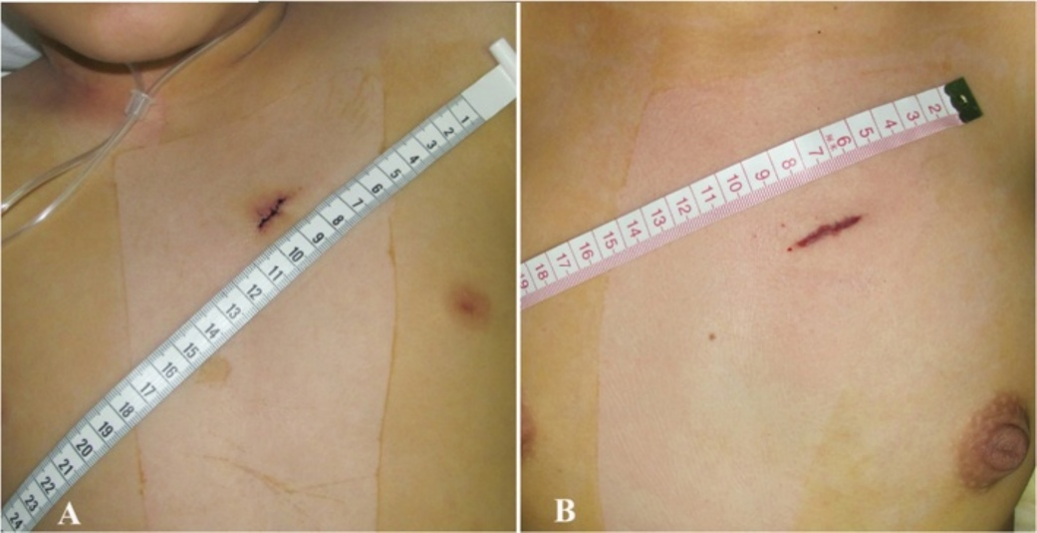
A 1.5 cm parasternal incision in a pediatric patient (**a**) and a 2.5 cm incision in an adult female patient (**b**)

A 1.5 cm to 3 cm parasternal incision was made in the left second or third intercostal space (within the “bikini lines” in female patients; Fig. 2). Superficial tissues were opened with blunt dissection without entering into the pleural space. Exposure was optimized with a mini-retractor. The pericardium was incised and cradled. The puncture site was chosen at the infundibular anterior wall of the right ventricle just below the pulmonary annulus. Two parallel pursestring sutures of 4–0 or 5–0 polypropylene were placed at this site.

After anticoagulation with heparin (100U/kg), the delivery sheath loaded with the device was inserted into the right ventricle through the puncture site. Usually, the sheath was perpendicular to the septum and directed towards the defect. Under continuous TEE guidance, it was advanced through the defect into the left ventricle. Then the device was deployed and released following the previous reports [4–8].

**Fig. 3:**
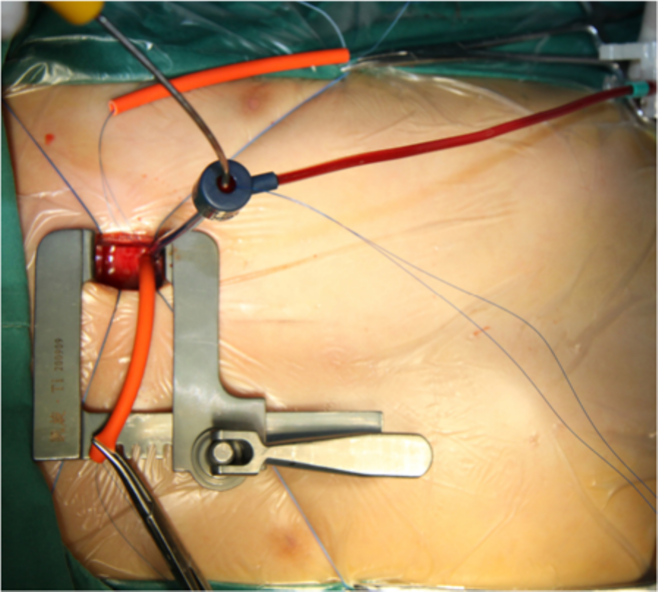
The delivery sheath loaded with the device was inserted into the right ventricle through the left second intercostal space. The side arm of the sheath was kept directing towards the apex during deployment

**Fig. 4:**
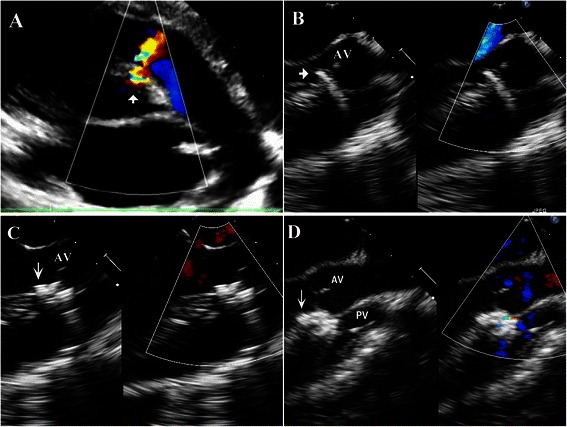
Different steps of perventricular device closure of a small doubly committed ventricular septal defect (DCVSD). **a** A small DCVSD (*arrowhead*) located at the 1 to 2 o’clock position in the parasternal short-axis view on transthoracic echocardiography. **b** The delivery sheath (*arrowhead*), which was perpendicular to the septum, was loaded with the device and passed through the defect. **c** The left disk was opened with its platinum marker (*arrow*) pointing towards the apex. While maintaining gentle tension on the cable without rotation, the delivery sheath was withdrawn to deploy the right disk of the device into the right ventricle. **d** The eccentric occluder was placed in a proper position without affecting the aortic and pulmonary valves. (AV = aortic valve, PV = pulmonary valve)

During the deployment of an eccentric occluder, the side arm of the sheath was kept directing towards the apex (Fig. 3). Then the platinum marker on the left disk was approximately pointing towards the apex. Figure 4 demonstrates the different steps of perventricular device closure of a DCVSD.

A complete TEE study was performed. The degree of a residual shunt was assessed by measuring the width of the color jet as previously reported [11]. The device was inspected repeatedly by a push-pull maneuver and released only when its proper position was obtained and interference with the aortic and pulmonary valve had been excluded. The device stay suture was kept for 15 min to observe the device location before it was removed. Then the sheath and cable were withdrawn with the pursestring sutures snugly tied. The pericardium was re-approximated without a drainage tube placement if the operative field was clean and dry. Otherwise, a central venous catheter used as a drainage tube was placed in the pericardium. The incision was closed in layers.

### Patient follow-up

Prophylactic antibiotics were started before the procedure and continued for 2 days. Most patients were discharged 3 to 5 days after the procedure and maintained on aspirin (3–4 mg · kg^−1^ · d^−1^) for the duration of 3 months. The follow-up protocol included assessments by electrocardiography and TTE at discharge, 1, 3, 6, 12 months and yearly after the procedure.

### Statistical analysis

All data were expressed as median (or mean ± standard deviation) and range. Intracardiac manipulation time was defined as the time the delivery sheath entered the right ventricle until the delivery sheath and cable were withdrawn from the right ventricle. The intracardiac manipulation time, procedure time, left ventricular end-diastolic diameter, postoperative hospital stay, defect and device size were recorded. Statistical comparisons of proportions were analyzed using a chi-square test (Stata10.0 software; StataCorp LP, College Station, TX). A probability value of less than 0.05 was defined as statistical significance.

## Results

### Intraoperative results

Successful device placement was achieved in 58/62 patients, with an immediate success rate of 94 %. Fifty-three of these patients had undergone this procedure through left second intercostal space except 4 adult patients through left third interspaces. The patient, who was associated with a mirror image dextrocardia, successfully underwent such a closure through the right second intercostal space. The procedural data and outcome for the successful patients are listed in Table 1.

Correct placement of the device at the first attempt was achieved in 44 patients (76 %). Redeployment of the device was necessary in 14 patients (24 %). Among them, the device was replaced with a smaller or eccentric occluder in 8 patients as a result of the encroachment on the aortic valve. The small eccentric was replaced with a larger one in 6 patients as a result of the unstable position.

The DCVSD failed to close in 4 patients (7 %). Two patients, one of whom had a bicuspid aortic valve, were switched to open repair as a result of device-related aortic regurgitation. The other two were converted to an open approach due to device migration and tilting after release.

### Postoperative and follow-up results

All successful patients were extubated within 2 h. Blood transfusion was not required in each case. Six cases of mild to moderate pericardial effusion occurred postoperatively because of no placement of a drainage tube. They all recovered after pericardiocentesis or taking diuretics 1 month later. Compared with the preoperative value, 48 patients had a decrease in left ventricular end-diastolic diameter at discharge (*p* = 0.073). Postoperative hospital stay was 4.3 ± 1.0 (range, 3–6) days.

All patients were followed up for a period of 180 to 1860 days (median 880 days) with TTE and electrocardiography—58 patients for 1 and 6 months, 42 for 1 year, 28 for 2 years, and 14 for 3 years. The follow-up rate was 100 %. Pre-existing aortic regurgitation remained unchanged or disappeared during the follow-up period. Device-related aortic regurgitation was not found. New mild pulmonary regurgitation occurred in 2 cases, which remained stable during the follow-up periods. Thrombosis, hemolysis, infective endocarditis or conduction abnormalities were not encountered. Of the successful 58 patients, 50 (86 %) had a complete closure, 8 (14 %) had a trivial or small residual shunt immediately after device release. The shunts disappeared during the follow-up period except one patient. At the last echocardiographic evaluation during follow-up, the complete closure rate was 95 % at discharge, 98 % at 1-, 6-, and 12-month, 96 % at 2-year and 100 % at 3-year (Table 2).

**Table 2:** Postoperative and follow-up results after successful device closure of doubly committed juxtaarterial ventricular septal defects

	IADR (*n* = 58)	At discharge (*n* = 58)	1 Months (*n* = 58)	6 Months (*n* = 58)	12 Months (*n* = 42)	24 Months (*n* = 28)	36 Months (*n* = 14)
Pre-existing AR (*n* = 16)	12	9	7	6	4	2	1
New AR (n)	0	0	0	0	0	0	0
New mild PR (n)	2	2	2	2	1	1	0
Complete Closure (%)	50/58 (86)	55/58 (95)	57/58 (98)	57/58 (98)	41/42(98)	27/28(96)	14/14(100)
Trivial RS (%)	6/58 (11)	1/58 (2)	0	0	0	0	0
Small RS (%)	2/58 (3)	2/58 (3)	1/58 (2)	1/58 (2)	1/42 (2)	1/28(4)	0
Follow-up median (days)			880 (180–1860)				

*IADR* immediately after device release; *AR* aortic regurgitation; *PR* pulmonary regurgitation; *RS* residual shunts

## Discussion

DCVSD is the least common type of VSD in the Western Hemisphere, accounting for approximately 5 % of such defects. But it is much more common in patients of Eastern Asian descent (about 25 % of Asian patients with a VSD). Its location near the aortic and pulmonary valves accounts for the unique features associated with this defect. Lack of support for the right aortic leaflet is crucial to the development of aortic valve prolapse or regurgitation. Moreover, spontaneous closure of this defect is not common. Therefore, numerous practitioners suggest that the DCVSD should be closed as soon as possible [12, 13].

### Perventricular versus percutaneous approach

Surgical repair remains the preferred treatment with good clinical outcomes. However, it is associated with morbidity, discomfort, and an unsightly scar. Cardiopulmonary bypass is needed, which is widely recognized as having a number of adverse effects.

Although percutaneous closure of VSD by different devices has been reported for more than 20 years with good results [1–3], it was mainly performed in patients with the perimembranous or muscular type of VSD. An arteriovenous guide-wire loop has to be established. Sometimes it is even impossible to perform the intervention, due to patient’s low weight or vascular access issues. Another important concern of percutaneous closure technique is the exposure to radiation which is associated with a spectrum of malignancy especially in children [14, 15].

In recent years, a number of reports on perventricular device closure of perimembranous VSDs have been published with encouraging results [4–7]. Compared with the percutaneous approach, the perventricular approach has the advantages of a short entry route, no weight and no age limitations, excellent manipulability of a short delivery sheath, no need to establish an arteriovenous guidewire loop, simple process of recapture and redeployment of the device, and no exposure to radiation.

In patients with a DCVSD, the spiraling course of the ventricular septum makes the percutaneous closure more difficult. Proper positioning of closure devices requires precise definition of septal and valvar anatomy, which is poorly defined by angiography. The perventricular technique is performed under TEE which is capable of imaging details of intracardiac anatomy as well as closure devices. The perventricular approach makes it possible for the sheath to pass through the defect easily and to position the device precisely.

### Advantages of the parasternal over inferior median sternotomy approach

1. Although the skin incision of the inferior median sternotomy is small, the length of underlying partial sternotomy is usually 3 to 5 cm longer than the length of skin incision. Bleeding due to the sternotomy is inevitable. A pericardial drainage tube is needed in each case. Postoperative pain and the pectus carinatum deformity are common after this procedure [7, 16]. The parasternal approach is performed through an intercostal access port without entering into the pleural space. In most cases, there is no need of a drainage tube. Therefore, the parasternal approach leads to less operative trauma, less pain, less blood loss and better cosmetic results.
2. The plane of the infundibular septum lies almost perpendicular to that of the remainder of the septum. The inferior partial sternotomy incision is unable to provide an entry route which is perpendicular to the infundibular septum unless the incision is extended superiorly to expose the infundibular wall of the right ventricle [8]. However, the left second intercostal access port faces directly towards the pulmonary annulus. During deployment of the device, the septum is approached from an anterior and not a lateral plane. This makes the DDS more perpendicular to the infundibular septum, resulting in easier defect crossing and accurate device positioning.
3. Once the perventricular closure fails, the patient is converted to a surgical repair directly. A reversed hockey stick incision is made by extending the original incision downward from the left second interspaces to the mid-sternum. The skin and subcutaneous tissue over the manubrium is mobilized and retracted for performing a full median sternotomy. The upper curved part of the skin incision, which can be covered by the low collar, also provides cosmetic results.

### Feasibility and safety

The DCVSD is near the aortic and pulmonary valve. Three important concerns are involved in the device closure technique: (1) the left disk might impinge on the aortic valve, (2) the right disk might affect the pulmonary valve, and (3) the device position might be unstable after release. Thus, in the previous reports [17], the DCVSD is not an indication for device closure.

The DDS, which is perpendicular to the septum and easy to manipulate, facilitates appropriate orientation of the eccentric occluder with regard to the aortic valve and cardiac apex. In the DCVSDs of less than 6 mm in diameter, the superior part of the left disk has no or relatively small contact area with the aortic valve. Thus the device does not interfere with the aortic valve. If a new aortic regurgitation were found after device positioning, the device would be recaptured and replaced with a small or eccentric one.

A DCVSD has no or a short muscular rim between the defect and the pulmonary valve annulus [17]. Usually, the small-sized DCVSD lies beneath commissure between right and left leaflets of the pulmonary valve. There is a subcommissural triangle between the right and left leaflets of the pulmonary valve, separating the right disk from the pulmonary valve. And the pulmonary annulus is a little bit higher than the aortic annulus. These decrease the risk of encroachment of the right disk on the pulmonary valve.

Although the aortic and pulmonary rim is deficient, the rest circumferential rim can provide enough support to the occluder in the small-sized DCVSDs. As described above, this approach has many additional advantages: the perpendicular short entry route, the ability to make fine adjustments in device position, the reliability to test device stability, and the ability to retrieve a suboptimally placed device. In order to avoid device embolization, the device stay suture is applied to all patients in our study. The suture has improved the safety of this technique. It helps to retrieve the device through a larger delivery sheath if the device is dislocated after release.

### Pitfalls with the parasternal approach

(1) Because the parasternal approach provides better operative exposure in pediatric than in adult patients, the ICMT (*p* > 0.05) and procedural time (*p* < 0.01) were shorter in the younger than in the older age group. (2) The incision cannot be made too close to the left border of the sternum to avoid injuring the left internal mammary artery [18]. The exact location of the incision is determined according to the chest film and TTE. The level of pulmonary annulus of adult patients seems lower than that of pediatric patients. Four adult patients underwent this procedure through the third intercostal space in this series. (3) Trivial or small residual shunts can be ignored in device closure technique as they usually disappear spontaneously during the follow-up period. (4) If the operative field is not clear and dry, a pericardial drainage tube is required to prevent pericardial effusion. (5) Pre-existing trivial or mild aortic regurgitation is not a contraindication for device closure of the DCVSDs. In some cases, it may disappear during the follow-up period.

### Study limitations

This study is not a prospective randomized study comparing the parasternal approach with inferior median sternotomy or percutaneous approach. We just speculated its safety and advantages according to the previous reports, our experience and results in our study. We reported only 58 successful patients using this technique and only 28 of them had been followed for more than 2 years. Further studies are required to establish long-term results in a larger patient population.

## Conclusions

Our study has demonstrated that perventricular device closure of a DCVSD with the size of less than 6 mm is feasible, safe and efficacious through a left parasternal approach. This minimally invasive technique permits easy defect crossing and accurate device positioning.

## Abbreviations

DCVSD: Doubly committed juxtaarterial ventricular septal defect
TEE: Transesophageal echocardiography/echocardiographic
TTE: Transthoracic echocardiography
DDS: Direct delivery system
VSD: Ventricular septal defect
